# Angiotensin II Type 1 Receptor Expression and Anti-AT1R Antibodies in Heart Transplantation: A Systematic Review of Distinct but Related Non-HLA Immune Pathways

**DOI:** 10.3390/jcm15145419

**Published:** 2026-07-10

**Authors:** Radha Gopalan, Mohamed Reyad Mohamed, Jamal Mahar, Anusha Sunkara, Anantharam Kalya, Abdelrahman Hafez, Nancy Reinsmoen, Francisco Arabia

**Affiliations:** 1 Cardiology Department, Banner University Medical Center, Phoenix, AZ 85006, USA; 2 Department of Medicine, University of Arizona College of Medicine-Phoenix, Phoenix, AZ 85004, USA; 3 Department of Cardiovascular Medicine, Mayo Clinic, Phoenix, AZ 85054, USA; 4 HLA and Immunogenetics Laboratory, Cedars-Sinai Medical Center, Los Angeles, CA 90048, USA

**Keywords:** heart transplantation, angiotensin II type 1 receptor, anti-AT1R antibodies, AT1R-Ab, Non-HLA antibodies, antibody-mediated rejection, acute cellular rejection, cardiac allograft vasculopathy, left ventricular assist device

## Abstract

**Background**: Heart transplantation (HT) remains the definitive therapy for end-stage heart failure, yet rejection and cardiac allograft vasculopathy (CAV) continue to limit long-term outcomes. Beyond donor-specific HLA antibodies, non-HLA antibodies, particularly anti-angiotensin II type 1 receptor antibodies (AT1R-Abs), have been implicated in allograft injury, but published findings are heterogeneous. **Aim**: The aim of this study is to systematically evaluate the evidence linking AT1R gene expression and anti-AT1R antibodies with key post-heart transplant outcomes. **Methods:** We conducted a systematic review in accordance with PRISMA guidelines. Scopus, PubMed, Web of Science, and the Cochrane Library were searched (December 2025) for cohort and case–control studies evaluating AT1R gene expression and/or AT1R-Ab status in HT recipients and their association with post-transplant outcomes. Two reviewers independently screened studies, extracted data, and assessed risk of bias using the NIH Quality Assessment Tool. **Results:** Twelve studies encompassing 951 recipients met the inclusion criteria. Five studies evaluated AT1R mRNA expression, reporting variable patterns: several observed reduced AT1R/AT2R transcription after transplantation without clear clinical correlation, whereas others associated higher donor or recipient AT1R expression with transplant coronary artery disease and recurrent rejection. AT1R-Ab prevalence varied widely and appeared to increase after mechanical circulatory support, with substantial seroconversion reported during LVAD support in initially antibody-negative patients. Associations between AT1R-Ab and acute cellular rejection and antibody-mediated rejection were inconsistent across studies, and survival findings were inconclusive; however, some reports linked elevated AT1R-Abs to poorer long-term freedom from adverse events. Evidence regarding CAV was mixed, with signals of increased vasculopathy risk in some cohorts but not others. **Conclusions**: Current evidence suggests a potential role for AT1R expression and AT1R-Abs in cardiac allograft dysfunction, including rejection phenotypes and vasculopathy. Larger prospective studies with harmonized testing strategies are needed to define clinically meaningful AT1R-Ab cutoffs and clarify their utility in risk stratification and targeted therapeutic trials.

## 1. Introduction

Heart transplantation (HT) serves as a definitive therapy for patients with end-stage heart failure (HF) who fail optimal medical treatment [1]. The survival rates post-HT are 85% at 1 year and 72% at 5 years [2]. The main challenges to survival after HT include nonspecific graft failure, acute or chronic rejection, and infection [3]. While rejection remains one of the major causes of heart graft dysfunction in the short term, cardiac allograft vasculopathy (CAV) is the most common cause in the long term [4]. Donor HLA-specific antibodies (DSAs) are associated with both etiologies for graft dysfunction and low survival after solid organ transplantation [1,2,3,4,5].

Graft rejection has also been linked to antibodies targeting donor non-HLA-specific and non-donor HLA-specific antigens [6,7]. Among the non-HLA antibodies, angiotensin II type 1 receptor antibodies (AT1R-Abs) are particularly significant as they are associated with graft rejection and transplant dysfunction [8]. Angiotensin II (Ang II), a main component of the renin–angiotensin–aldosterone system (RAAS), affects tissues by activating AT1R and AT2R receptors, leading to increased sympathetic activity, vasopressin release, and aldosterone production [9]. Ang II primarily affects tissue through AT1R in adults, leading to myocyte hypertrophy and hyperplasia [10]. Although the function of AT2R is less understood, its high expression during embryonic and neonatal stages suggests a role in growth regulation [11]. Additionally, AT2R activation inhibits the growth of vascular smooth muscle and endothelial cells and triggers apoptosis [12], suggesting that AT1R promotes growth and AT2R may exert inhibitory effects.

Anti-AT1R antibodies can arise via various routes, including autoreactive reactions brought by pregnancy, transplantation, transfusion, stopping immunosuppressive medication, or noncompliance [13]. Moreover, studies have shown that mechanical cardiac support device implantation significantly increases AT1R antibody levels [14,15]. Anti-AT1R-Abs are linked to various conditions, including intrauterine growth retardation [16,17], eclampsia [18], systemic sclerosis [19], early renal [20,21], liver [22], lung [23] transplant loss as well as Alzheimer’s disease [24]. Additionally, it has been shown that not only are the AT1R antibodies linked to cardiac graft dysfunction, but patients with CAV exhibit higher expression of the AT1R gene [25,26].

Although the relationship between AT1R-Abs and renal graft dysfunction has been established [27], emerging evidence suggests a clinically relevant role for AT1R expression and AT1R-Abs in cardiac allograft dysfunction. However, existing studies report heterogeneous and occasionally conflicting findings, and the underlying mechanisms and prognostic implications remain incompletely understood [16,25,28,29,30,31,32,33].

This review, a systematic narrative synthesis, aims to complement present knowledge gaps regarding the effects of AT1R and AT1R-Abs on cardiac graft outcomes by summarizing and clarifying their effects before and after HT.

## 2. Methods

We followed the Preferred Reporting Items for Systematic Reviews and Meta-analysis guidelines when conducting this systematic review [34]. A completed PRISMA 2020 checklist is provided in Supplementary Table S3. The review protocol was not registered. Because the review had already progressed to study screening and data extraction before protocol registration was considered, retrospective registration was not pursued to avoid presenting the protocol as prospectively registered. A list of excluded full-text articles with the reason for exclusion is provided in Supplementary Table S4.

### 2.1. Literature Search

The following search strategy ((“Angiotensin II Type 1 Receptor” OR “Angiotensin AT1 Receptor” OR “Angiotensin Type 1 Receptor” OR “Angiotensin II Type 1b Receptor” OR “Angiotensin Type 1b Receptor” OR “Angiotensin AT1b Receptor” OR “Angiotensin II Type 1a Receptor” OR “Angiotensin AT1a Receptor” OR “Angiotensin Type 1a Receptor” OR AT1R OR “Angiotensin II Receptor” OR “anti-AT1R” OR “AT1R-Ab” OR “AT1R antibody” OR “angiotensin II type 1 receptor antibody”) AND (“Heart Grafting*” OR “Heart Transplantation*” OR “Cardiac Transplantation*” OR “heart transplant” OR “cardiac allograft” OR “orthotopic heart transplantation”)) was used in December 2025 to search all relevant published articles at four electronic databases: Scopus, PubMed, Cochrane Library databases, and Web of Science. The full database-specific search strategies are provided in Supplementary Table S1. No study design restrictions were applied during the initial database search. However, only full-text articles published in English were eligible for inclusion during the screening and eligibility assessment. We checked the references for the included studies to make sure no citations were missing or added in error.

### 2.2. Eligibility Criteria

We included observational human studies, including casecontrol studies, prospective cohort studies, retrospective cohort studies, and retrospective analyses, that evaluated the pre- and post-transplant AT1R expression and/or compared clinical outcomes between AT1R-Ab-positive and AT1R-Ab-negative heart transplant patients. Eligible studies were required to include heart transplant recipients and report at least one clinically relevant post-transplant outcome, including acute cellular rejection, antibody-mediated rejection, cardiac allograft vasculopathy, graft dysfunction, survival, freedom from adverse events, or transplant coronary artery disease progression. We excluded editorials, reviews, letters, case reports/series, conference abstracts, research involving animals, duplicate publications, full text not being available, non-English studies, and non-HT studies.

### 2.3. Study Selection and Data Extraction

Two authors (MM and AH) evaluated the articles’ titles and abstracts separately to determine which publications to include. After that, they examined the entire texts of the selected articles, and disagreements were resolved by discussion and agreement. Data extraction followed standardized criteria, encompassing the first author’s name, year of publication, country of origin, number of transplant participants, mean age of recipients, study design, participant demographics, study conclusions, and primary outcomes.

### 2.4. Quality Assessment

Two impartial reviewers, MM and AH, evaluated bias using the National Institutes of Health (NIH) Quality Assessment Tool. To assess the internal validity of cross-sectional and cohort studies, including hazards like selection bias, information bias, measurement bias, or confounding, this tool consists of a checklist with 14 questions. Response choices of “yes,” “no,” “not applicable,” or “not reported” were used to assess each category. Because the NIH Quality Assessment Tool is intended primarily for qualitative domain-based appraisal and does not prescribe a formal numerical scoring system, we applied an author-defined, prespecified modified quantitative summary to improve transparency and reproducibility of item-level assessments. Responses were summarized as follows: Yes = 1 point, No = 0.5 points, and NR/NA/CD = 0 points. Studies were categorized as good (11–14 points), fair (7.5–10.5 points), or poor (0–7 points). This score was used only as an author-defined summary aid and should not be interpreted as a validated NIH scoring system. Final quality ratings were interpreted qualitatively in the context of the NIH domains, with particular attention to selection bias, exposure and outcome measurement, follow-up, and confounding [35]. Disagreements were resolved by discussion and consensus. The complete risk-of-bias assessment is provided in Supplementary Table S2.

## 3. Results

### 3.1. Study Selection

**Figure 1:**
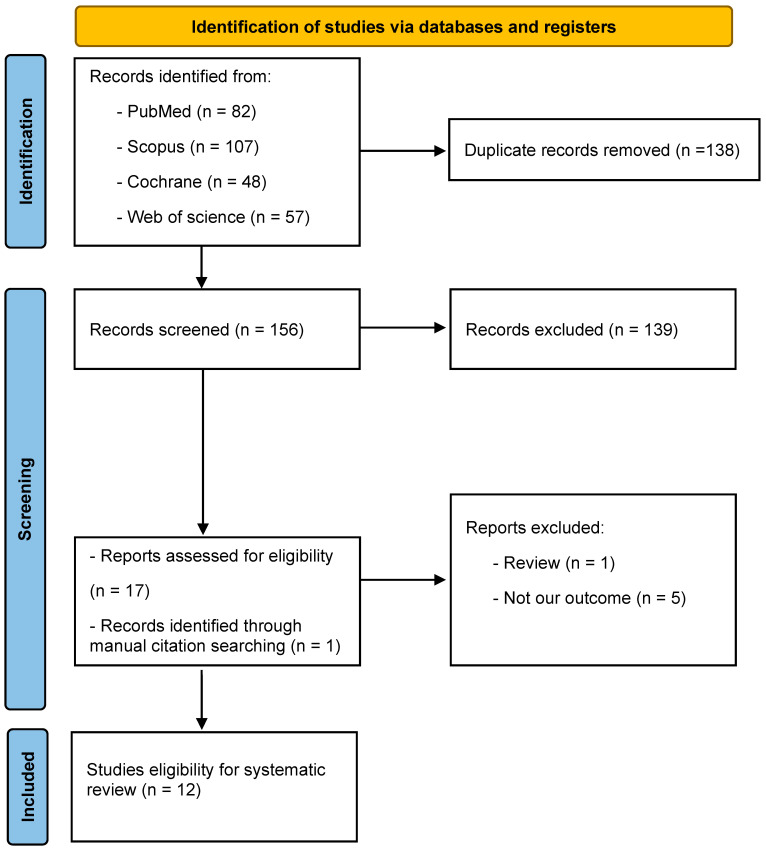
PRISMA 2020 flow diagram of study selection. The literature search identified 294 records from PubMed (*n* = 82), Scopus (*n* = 107), Cochrane Library (*n* = 48), and Web of Science (*n* = 57). After removal of 138 duplicate records, 156 records were screened, of which 139 were excluded. Seventeen full-text reports were assessed for eligibility, and six were excluded because they were review articles (*n* = 1) or did not report outcomes relevant to the review question (*n* = 5). A total of 12 studies were included in the systematic review. The excluded full-text articles and reasons for exclusion are summarized in Supplementary Table S4.

The database search identified 294 records, including 82 from PubMed, 107 from Scopus, 48 from Cochrane Library, and 57 from Web of Science. After removal of 138 duplicates, 156 unique records underwent title and abstract screening, of which 139 were excluded. Seventeen full-text reports were assessed for eligibility, and six were excluded: one was a review article and five did not report outcomes relevant to the review question. Eleven studies were included from database searching [16,25,26,28,29,30,36], and one additional eligible study was identified through manual citation searching [36], resulting in 12 studies included in the systematic review. The selection process is illustrated in the PRISMA flow diagram (Figure 1).

### 3.2. Baseline Characteristics

Across the 12 included studies (2 case–control, 1 cohort, 5 retrospective cohort, 3 prospective cohort, and 1 retrospective analysis), most were conducted in the USA. In total, 951 participants were represented across study arms, with several studies focusing on clinically defined subgroups (e.g., vasculopathy vs non-vasculopathy, AT1R-Ab positive vs controls, recurrent acute rejection vs controls, and LVAD-bridged recipients stratified by AT1R-Ab levels). Where reported, mean recipient age across arms ranged from 43.0 to 58.5 years, donor mean age ranged from 29.0 to 41.2 years, and the proportion of males ranged from 54.6% to 87.0%. Total ischemic time (reported in a subset of studies) had mean values spanning 170 to 217 min. The etiology of heart failure was variably described; in studies reporting it, ischemic cardiomyopathy comprised approximately 10% to 72.7% of recipients (with the remainder predominantly non-ischemic cardiomyopathy). The characteristics of the included studies are summarized in Table 1 and Table 2.

### 3.3. Risk-of-Bias Assessment

Using the NIH Quality Assessment Tool for observational cohort/cross-sectional studies, as summarized in Supplementary Table S2, the overall methodological quality of the included studies was mostly good, with 8/12 rated “good” and 4/12 rated “fair”; no study was rated “poor”. The ratings were based on a prespecified modified quantitative summary of NIH item-level responses, followed by qualitative interpretation of the major NIH domains. The most frequent limitations driving “fair” ratings were potential underpowering/small sample sizes, incomplete reporting or handling of key confounders (confounding was often not reported or not clearly adjusted for), and limited information on follow-up/attrition in a minority of studies [26,29,30,36].

### 3.4. AT1R Gene Transcription

From the studies included, five studies investigated the association between heart transplants and the expression of AT1R mRNA [25,26,29,33,36]. Varying patterns and associations with transplant outcomes were reported. Zagrosek et al., in a comparison of 16 orthotopic heart transplant (OHT) patients and 12 controls, observed a reduction in AT1 and AT2 mRNA post-transplant with no significant correlation between AT1R mRNA levels and clinical outcomes [36]. Similarly, Gullestad et al., comparing 17 OHT patients with ten donor hearts, confirmed a marked reduction in AT1 and AT2 mRNA [29]. Yousufuddin et al., through two studies, linked elevated AT1R mRNA levels in donors with a higher risk of transplant coronary artery disease (TCAD), finding early upregulation predictive of increased vasculopathy [26,33]. Yamani et al. associated recurrent rejection episodes with a progressive increase in AT1R mRNA, which remained high after resolution [25]. Additionally, Hiemann et al. demonstrated that AT1R-targeting antibodies correlated with increased AT1R mRNA, cellular and antibody-mediated rejection, and microvasculopathy, underscoring the receptor’s role in transplant pathology [38]. These findings suggest that mRNA expression of Angiotensin II receptors, particularly AT1R, plays a significant role in the pathophysiology of heart transplant rejection and related complications.

### 3.5. Prevalence of AT1R-Ab in Heart Transplant

In studies examining the prevalence of AT1R-Abs in heart transplant recipients, notable variation was observed. Hiemann et al. reported that 53% of patients exhibited elevated AT1R-Ab levels within the first-year post-transplant [38]. Urban et al. found 11.6% of patients had anti-AT1R antibodies before Heart Mate II LVAD implantation, with 63.8% of initially negative patients developing AT1R-Ab positivity during LVAD support [32]. Similarly, Chau et al. noted an increase in AT1R-Ab from baseline following LVAD implantation, with 59.7% of initially negative patients developing antibodies before subsequent transplantation [37]. Thohan et al. detected anti-AT1R antibodies in 57% of patients pre-transplant and 49% following transplantation, highlighting a persistence of AT1R-Ab in nearly half of the recipients [31]. Given the heterogeneity in AT1R-Ab testing and outcome definitions across studies, key methodological and clinical variables are summarized in Table 3. These include assay types, cutoff values, timing of antibody measurement, LVAD/MCS exposure, HLA-DSA or HLA antibody contexts, immunosuppression reporting, rejection definitions, and CAV definitions.

### 3.6. AT1R-Ab and Acute Cellular Rejection

Studies on the relationship between acute cellular rejection (ACR) and AT1R-Ab status in heart transplant patients show mixed results. Thohan et al. and Urban et al. reported no significant difference in ACR incidence based on anti-AT1R status, with similar rates of freedom from ACR at one year between anti-AT1R-negative and -positive groups [31,32]. Conversely, Hiemann et al. and Chau et al. found that elevated AT1R-Ab levels were associated with increased cellular rejection rates [37,38]. These findings highlight variability in the ACR risk associated with anti-AT1R antibodies.

### 3.7. AT1R-Ab and Antibody-Mediated Rejection

The relationship between AT1R-Ab and AMR in heart transplant patients shows variability across studies. Thohan et al. and Urban et al. found no significant difference in AMR frequency based on anti-AT1R status [31,32]. In contrast, Chau et al. observed that elevated AT1R-Ab levels were associated with a higher rate of AMR, with a hazard ratio (HR) of 3.8, but this was statistically insignificant (95% CI: 0.4–34.9, *p* = 0.23) [37]. Similarly, Reinsmoen et al. reported that high levels of AT1R-Ab were linked to reduced freedom from AMR, although this result was also statistically nonsignificant (HR = 1.4, 95% CI: 0.5–3.6, *p =* 0.51) [16]. These findings indicate that while there is an observed association, the evidence for a significant impact of AT1R-Ab on AMR remains inconclusive.

### 3.8. AT1R-Abs and Survival

The impact of AT1R-Abs on survival in heart transplant patients is inconclusive. Thohan et al. found that the presence of anti-AT1R antibodies, either pre- or post-transplant, did not significantly affect 10-year survival rates (*p =* 0.061) [31]. Similarly, Urban et al. reported no significant difference in one- and five-year survival rates between anti-AT1R antibody-negative and antibody-positive patients (*p* = 0.582) [32]. Reinsmoen et al. did not specifically assess overall survival based solely on anti-AT1R status but indicated that the presence of dnDSA and high levels of AT1R-Abs were associated with reduced freedom from AMR and CMR HR = 8.0 (95% CI: 2.3–27.9, *p* < 0.0001) [16]. In contrast, Chau et al. found that elevated AT1R-Ab levels at the time of transplant were associated with poorer outcomes, including a lower rate of freedom from adverse events at five years (43%, log-rank *p* = 0.005) [37]. These findings suggest that while the effect of AT1R-Abs on survival is not universally significant, it may influence long-term transplant outcomes and rejection rates.

### 3.9. Cardiac Allograft Vasculopathy

The relationship between AT1R-Ab and CAV shows mixed findings across studies. Thohan et al. reported no significant impact of AT1R-Ab presence, either pre- or post-transplant, on CAV incidence [31]. Similarly, Reinsmoen et al. did not identify a statistically significant association, though recipients with elevated post-transplant AT1R-Ab levels had a higher, albeit nonsignificant, incidence of CAV [16]. In contrast, Yousufuddin et al. demonstrated a significant correlation between elevated AT1R mRNA levels in donor hearts and TCAD progression, with notable associations between donor spleen lymphocyte AT1R mRNA levels and CMIT and plaque volume one week (r = 0.52, *p* < 0.005) and one-year post-transplant (r = 0.63, *p* < 0.0001) [33]. Another study by Yousufuddin et al. linked transplant vasculopathy with higher cardiac AT1R expression (*p* = 0.006) [26]. Chau et al. found elevated AT1R-Ab levels after LVAD implantation were associated with adverse post-transplant events, including CAV [37]. Additionally, Hiemann et al. reported a significant relationship between AT1R and CAV incidence (*p* = 0.048) [38]. These findings suggest AT1R expression and antibodies may influence CAV development, though further research is needed for definitive conclusions.

## 4. Discussion

This systematic review summarizes the available heart-transplant literature evaluating two related but methodologically distinct non-HLA immune signals: myocardial or donor-tissue AT1R expression and circulating anti-AT1R antibodies. The principal finding is that both evidence streams suggest biologic plausibility for involvement in allograft injury, but the clinical evidence remains inconsistent and limited by heterogeneity in study design, assay methods, timing of assessment, outcome definitions, and adjustment for important confounders. Among the included heart-transplant studies evaluating AT1R expression, reported associations were variable. Zagrosek et al. and Gullestad et al. reported reduced AT1R and AT2R mRNA expression after transplantation without a consistent relationship with clinical outcomes [29,36]. In contrast, Yousufuddin et al. linked higher cardiac or donor-derived AT1R expression with subsequent transplant coronary artery disease, and Yamani et al. reported increased AT1R expression among recipients with recurrent rejection [25,26,33]. Hiemann et al. further connected receptor-targeting antibodies, increased AT1R mRNA expression, rejection, and microvasculopathy [38]. These findings support a potential role for local receptor expression in vascular and inflammatory allograft pathology, but they should not be interpreted as equivalent to circulating anti-AT1R antibody positivity.

The evidence from included anti-AT1R antibody studies is also mixed. Some cohorts reported associations between elevated anti-AT1R antibodies and cellular rejection, antibody-mediated rejection, microvasculopathy, CAV, or composite adverse outcomes [16,30,38]. Other studies found no statistically significant relationship between anti-AT1R antibody status and acute cellular rejection, antibody-mediated rejection, CAV, or survival [31,32]. These conflicting findings may reflect true biologic heterogeneity, but they may also reflect differences in antibody assay platform, positivity threshold, pre- versus post-transplant sampling, LVAD exposure, coexistence of HLA donor-specific antibodies, immunosuppression, follow-up duration, and nonuniform definitions of rejection and CAV.

Mechanical circulatory support is an important interpretive modifier. Studies of patients bridged to transplantation with continuous-flow LVADs reported development or amplification of anti-AT1R antibodies during LVAD support, with some analyses linking higher antibody levels to worse post-transplant outcomes [32,37]. Therefore, anti-AT1R positivity in LVAD-bridged recipients should be interpreted in the context of device-associated immune activation, pre-existing sensitization, HLA antibody status, and the timing of antibody measurement relative to device implantation and transplantation [39].

Mechanistic and non-heart-transplant literature provides useful context but should be distinguished from the evidence included in this heart-transplant systematic review. Experimental studies suggest that activating anti-AT1R antibodies can stimulate AT1R-dependent signaling, promote endothelial activation, increase inflammatory mediators, and contribute to vascular injury [20,40,41,42]. Kidney, liver, lung, and pediatric case-report literature further supports the broader concept that non-HLA antibodies may participate in allograft injury [8,20,21,22,23,43,44,45,46]. However, these data are indirect when applied to adult heart transplantation and should not be used as direct evidence of clinical effect in heart transplant recipients.

Therapeutic implications should also be interpreted cautiously. Angiotensin receptor blockers, plasmapheresis, IVIG, steroids, and other desensitization or anti-inflammatory strategies have been described in mechanistic studies, kidney transplantation, case reports, and small retrospective experiences [47,48,49,50,51,52,53,54]. These approaches remain hypothesis-generating for heart transplantation. Current evidence does not establish that anti-AT1R antibody testing alone should dictate treatment, nor does it define an optimal antibody threshold, treatment trigger, ARB choice, or monitoring strategy in heart transplant recipients.

Several limitations should be acknowledged. The included studies were generally small, observational, and heterogeneous with respect to AT1R-Ab assay methods, positivity thresholds, timing of measurement, rejection and CAV definitions, follow-up duration, and adjustment for confounders. Risk-of-bias assessment further limited the strength of inference, as several studies were rated as fair because of incomplete confounder adjustment, limited sample size, and incomplete reporting of follow-up or attrition. Another important limitation is the inconsistent reporting of RAAS-modulating therapies, including ACE inhibitors, ARBs, and MRAs. These agents may plausibly modify AT1R-mediated signaling and could therefore confound the association between AT1R-Ab status and post-transplant outcomes; however, their use, timing, dose, and indication were not systematically reported across the included studies.

Future studies should prospectively evaluate standardized anti-AT1R assays, prespecified thresholds, uniform sampling times, concurrent HLA-DSA status, LVAD exposure, immunosuppressive regimens, RAAS-modulating therapies, and harmonized definitions of AMR, ACR, microvasculopathy, and CAV. Until such data are available, AT1R expression and anti-AT1R antibodies should be viewed as related but distinct biologic signals with possible relevance to allograft injury, rather than as validated standalone clinical biomarkers.

## 5. Conclusions

In summary, the available heart-transplant literature suggests that AT1R expression and anti-AT1R antibodies may be associated with rejection phenotypes, microvascular injury, CAV/TCAD, and adverse graft outcomes in selected cohorts. However, current evidence remains limited by small observational cohorts and substantial methodological heterogeneity. AT1R expression and circulating anti-AT1R antibodies should therefore be interpreted as related but distinct biological signals, not as interchangeable or validated standalone clinical biomarkers. Larger prospective studies using standardized assays, prespecified thresholds, uniform rejection and CAV definitions, concurrent HLA/non-HLA antibody profiling, and careful adjustment for LVAD exposure, HLA-DSA status, immunosuppression, and RAAS-modulating therapies are required before definitive clinical recommendations can be made.

## Figures and Tables

**Table 1:** Characteristics of included studies evaluating AT1R expression and AT1R antibodies in heart transplantation.

No.	Study	Study Design	Country	Inclusion Criteria	Primary Endpoint(s)	Key Conclusion
1	Zagrosek et al., 1996 [36]	Case–control study	Germany	Heart transplant recipients undergoing diagnostic cardiac catheterization; all had normal right and left ventricular ejection function.	AT1 mRNA copies in endomyocardial biopsies from normal and transplanted human hearts.	Ventricular AT1 mRNA was downregulated after orthotopic heart transplantation. The decrease was not associated with systolic function and may partially reflect loss of autonomic nerves and altered nervous control of the heart.
2	Gullestad et al., 1998 [29]	Case–control study	Norway	Heart transplant recipients studied either 1 week or >1 year after transplantation.	Expression levels of angiotensin II receptor subtypes AT1 and AT2 mRNA in myocardial biopsies; comparison between early and annual post-transplant evaluation.	mRNA for both angiotensin II receptors was downregulated after heart transplantation. Post-transplant hypertrophy did not appear to be driven by increased transcription of the AT1 receptor.
3	Yousufuddin et al., 2004a [26]	Cohort study	USA	Heart transplant recipients who underwent serial surveillance endomyocardial biopsies through the first post-transplant year.	Expression of AT1R and AT2R mRNA in lymphocytes and donor hearts at 1 week and 1 year after transplantation; average biopsy score; changes in maximal intimal thickness and plaque volume over 1 year.	The data suggested a role for angiotensin II receptors in TCAD pathogenesis and supported the concept that TCAD may originate in the donor and be modified by recipient biological factors.
4	Moreno et al., 2022 [30]	Retrospective cohort study	USA	OHT recipients from 2017 to 2019 who presented with heart failure symptoms or imaging evidence of graft dysfunction, including LVEF drop >10%, LVEF ≤5%, or new diastolic dysfunction.	AT1R-Ab levels and their potential association with nonspecific graft dysfunction.	AT1R-Ab-mediated rejection in OHT remains poorly understood. More than half of patients with graft dysfunction in the absence of acute cellular rejection or canonical AMR had elevated AT1R-Ab titers.
5	Yousufuddin et al., 2004b [33]	Retrospective cohort study	USA	Heart transplant recipients who underwent surveillance right ventricular endomyocardial biopsies and IVUS studies within the first post-transplant year.	AT1R and AT2R mRNA transcript levels; changes in maximal intimal thickening and plaque volume by IVUS; average biopsy score for cellular rejection.	Cardiac angiotensin II receptor gene transcripts were associated with TCAD progression. AT1R gene transcripts predicted transplant vasculopathy in this preliminary study.
6	Chau et al., 2020 [37]	Retrospective cohort study	USA	LVAD patients who underwent primary heart transplantation between January 2009 and December 2017, with follow-up data through May 2019; no patients underwent antibody desensitization before or after transplantation.	Impact of AT1R-Ab levels on post-transplant outcomes in LVAD-bridged recipients; freedom from adverse events defined as death, treated rejection, or CAV grade ≥2.	In LVAD-bridged patients, elevated AT1R-Ab at transplant was associated with poorer post-transplant outcomes. Post-LVAD AT1R-Ab elevations did not correlate with systemic inflammatory markers.
7	Yamani et al., 2006 [25]	Retrospective cohort study	USA	Heart transplant recipients with recurrent acute rejection, defined as three consecutive episodes of acute cellular rejection grade 3A or higher, matched to controls without rejection in the first post-transplant month.	AGTR1 mRNA expression during rejection episodes; impact of ACE polymorphism on rejection.	AGTR1 mRNA expression increased during recurrent cellular rejection and decreased after treatment, but did not completely return to baseline.
8	Reinsmoen et al., 2014 [16]	Retrospective archived-serum cohort	USA	Heart transplant recipients transplanted from May 2007 to August 2011 with pre- and post-transplant sera available for DSA and AT1R-Ab testing.	Impact of DSA and AT1R-Ab at transplant and post-transplant de novo DSA/AT1R-Ab on heart allograft outcomes.	Combined de novo DSA and AT1R-Ab had a greater negative impact on freedom from AMR and/or CMR than either marker alone.
9	Hiemann et al., 2012 [38]	Prospective cohort study	Germany	De novo HT recipients transplanted between September 2005 and October 2006.	Presence of non-HLA antibodies, including AT1R-Ab and ETAR-Ab; incidence of microvasculopathy and acute rejection during the first post-transplant year.	Elevated AT1R-Ab and ETAR-Ab levels were associated with cellular rejection, antibody-mediated rejection, and early microvasculopathy.
10	Dieterlen et al., 2014 [28]	Cross-sectional cohort study	Germany	HT recipients with an up-to-date coronary angiogram, known donor-HLA status, and antibody diagnostics.	Role of antibodies against non-HLA antigens, including MICA, AT1R, and ETAR, in CAV; identification of recipients at risk for CAV.	HLA and non-HLA antibodies were associated with CAV after HT. Non-HLA antibodies were linked to earlier and higher CAV incidence.
11	Thohan et al., 2020 [31]	Retrospective cohort study	USA	Adult cardiac transplant recipients with historical serum samples from final crossmatch and, when available, saved post-transplant serum samples from routine clinical practice.	Long-term patient survival and cardiac function according to AT1R-Ab status.	AT1R-Ab detected before or after heart transplantation was not associated with clinically important outcomes, including LVEF, ACR, AMR, CAV, or overall survival.
12	Urban et al., 2016 [14]	Prospective serum evaluation with retrospective outcome analysis	Czech Republic	Patients implanted with HeartMate II between October 2008 and August 2014 who subsequently underwent heart transplantation.	Post-transplant survival and freedom from acute cellular and antibody-mediated rejection in HeartMate II-bridged recipients stratified by pretransplant AT1R-Ab status.	No difference was observed in post-transplant survival or freedom from acute cellular or antibody-mediated rejection between AT1R-Ab-negative and AT1R-Ab-positive recipients.

Abbreviations: ACR, acute cellular rejection; AGTR1, angiotensin II receptor type 1 gene; AMR, antibody-mediated rejection; AT1R, angiotensin II type 1 receptor; AT1R-Ab, angiotensin II type 1 receptor antibody; AT2R, angiotensin II type 2 receptor; CAV, cardiac allograft vasculopathy; CMR, cellular-mediated rejection; DSA, donor-specific antibody; ETAR, endothelin type A receptor; HLA, human leukocyte antigen; HT, heart transplantation; IVUS, intravascular ultrasound; LVAD, left ventricular assist device; LVEF, left ventricular ejection fraction; MICA, major histocompatibility complex class I chain-related antigen A; OHT, orthotopic heart transplantation; TCAD, transplant coronary artery disease.

**Table 2:** Baseline demographic and clinical characteristics of study participants across included studies.

No.	Study	Study Arm, *n* (%)	Recipient Age, Mean ± SD, Years	Donor Age, Mean ± SD, Years	Male, *n* (%)	Induction Immunosuppression, *n* (%)	Total Ischemic Time, Mean ± SD, min	Etiology of Heart Failure *	
								ICM, *n* (%)	NICM, *n* (%)
1	Zagrosek et al., 1996 [36]	Heart transplant recipients, 16 (57.1)	48.1 ± 14	NA	NR	NR	NA	NR	NR
		Control group, 12 (42.9)	43.7 ± 14	NA	NR	NR	NA	NR	NR
2	Gullestad et al., 1998 [29]	1-week evaluation group, 6 (35.3)	49 ± 15	29 ± 12	5 (83.3)	NR	217 ± 44	4 (66.7)	2 (33.3)
		Annual evaluation group, 11 (64.7)	43 ± 8	NR	9 (81.8)	NR	NR	8 (72.7)	3 (27.3)
3	Yousufuddin et al., 2004a [26]	Vasculopathy group, 15 (53.6)	56.9 ± 9.9	39.8 ± 12.2	10 (66.7)	NR	170 ± 53	NR	NR
		Non-vasculopathy group, 13 (46.4)	56.0 ± 12.2	30.4 ± 11.9	9 (69.2)	NR	NR	NR	NR
4	Moreno et al., 2022 [30]	AT1R-Ab group, 11 (52.4)	52.81 ± 17.38	NR	6 (54.5)	1 (9.1)	NR	3 (27.3)	8 (72.7)
		Control group, 10 (47.6)	49.1 ± 12.31	NR	6 (60.0)	NR	NR	1 (10.0)	9 (90.0)
5	Yousufuddin et al., 2004b [33]	Overall cohort, 45 (100)	56 ± 13	35 ± 14	NR	NR	171 ± 53	NR	NR
6	Chau et al., 2020 [37]	Normal post-LVAD AT1R-Ab, 31 (40.3)	56.67 ± 13.21	NR	23 (74.2)	13 (42.0)	204.33 ± 53.63	NR	NR
		Elevated post-LVAD AT1R-Ab, 46 (59.7)	52 ± 13.77	NR	34 (73.9)	23 (50.0)	208.33 ± 50.51	NR	NR
7	Yamani et al., 2006 [25]	Recurrent acute rejection group, 14 (48.3)	53 ± 14	33 ± 15	9 (64.0)	2 (14.3)	174 ± 47	7 (50.0)	6 (42.9)
		Control group, 15 (51.7)	55 ± 13	31 ± 14	9 (60.0)	3 (20.0)	187 ± 48	8 (53.3)	6 (40.0)
8	Reinsmoen et al., 2014 [16]	Heart transplant recipients, 200 (100)	NR	NR	NR	NR	NR	NR	NR
9	Hiemann et al., 2012 [38]	Heart transplant recipients, 30 (100)	48	41.2 ± 2.2	22 (73.3)	30 (100)	208.8 ± 9.6	NR	17 (56.7)
10	Dieterlen et al., 2014 [28]	Heart transplant recipients, 116 (100)	58.5 ± 12.8	40.35 ± 13.37	91 (78.4)	116 (100)	NR	33 (28.4)	72 (62.1)
11	Thohan et al., 2020 [31]	Heart transplant recipients, 291 (100)	55 ± 11	NR	218 (74.9)	<2%	NR	157 (54.0)	124 (42.6)
12	Urban et al., 2016 [14]	Heart transplant recipients, 69 (100)	49.47 ± 14.73	NR	60 (87.0)	69 (100)	NR	23 (33.3)	NR

Data are presented as mean ± SD or n (%) as reported in the original studies. Empty or unavailable fields were standardized as NR or NA. Abbreviations: AT1R-Ab, angiotensin II type 1 receptor antibody; ICM, ischemic cardiomyopathy; LVAD, left ventricular assist device; NA, not applicable; NICM, nonischemic cardiomyopathy; NR, not reported; SD, standard deviation. * Percentages for ICM and NICM may not sum to 100% because other etiologies were present or not separately reported.

**Table 3:** Methodological and clinical heterogeneity among included studies directly measuring AT1R antibodies in heart transplantation. Only studies directly measuring AT1R-Abs were included. Studies limited to AT1R/AGTR1 mRNA or receptor-expression measurements without AT1R-Ab testing were excluded.

Study	Assay Type and AT1R-Ab Cutoff	Timing of Measurement	LVAD/MCS Exposure	HLA-DSA/HLA Antibody Context	Immunosuppression	Clinical Definitions: Rejection/CAV
Hiemann et al., 2012 [38]	Cell-based ELISA, CellTrend. ROC cutoff: 15.9 U/L for combined ACR/graft-failure endpoint; high vascular-receptor antibody status also reported as maximum AT1R-Ab >16.5 U/L during follow-up.	Serial post-HT testing at 24 h, 2 wk, 1 mo, 6 mo, and 1 yr. Highest titers within 24 h were interpreted as compatible with preformed sensitization.	14/30 (47%) bridged with MCS: LVAD 9, BiVAD 5.	Pretransplant HLA class I/II antibodies assessed; HLA class I antibodies in 4/30 (13%) and class II antibodies in 2/30 (7%). HT performed after negative crossmatch; no de novo HLA antibodies during follow-up.	ATG induction 1.5 mg/kg on days 0 and 1; cyclosporine A plus everolimus or MMF plus steroid taper.	ACR assessed by ISHLT histology. AMR assessed by immunohistochemistry in CD31-positive capillaries with CD68, C3d, IgA, IgM, and IgG positivity. Microvasculopathy defined as luminal radius/wall diameter ratio <1 in at least one vessel per field. Epicardial vasculopathy assessed by 1-year angiography and classified by ISHLT.
Dieterlen et al., 2014 [28]	ELISA, CellTrend, using native receptor conformation immobilized on the solid phase. ROC-derived AT1R cutoff: 8.32 U/mL; logistic regression table reports 8.325 U/mL.	Single posttransplant antibody screening during routine long-term follow-up.	NR.	HLA/MICA screened by LABScreen Mixed; positive sera further tested by single-antigen assay. MFI cutoff for DSA/specific antibody characterization: 500. DSA reported in 8 recipients.	ATG induction 1 mg/kg on day 0; triple regimen with CNI plus MMF or azathioprine plus corticosteroids.	BPAR graded by ISHLT 1990/2004 criteria. Grade 1A was not treated; grade 1B/1R or higher was treated. No histologic evidence of AMR by C4d criterion. CAV diagnosed by coronary angiography using ISHLT standardized nomenclature; protocol angiography started 1 year after HT and was repeated every other year if no CAV was detected.
Reinsmoen et al., 2014 [16]	Sandwich ELISA using native AT1R configuration, CellTrend/One Lambda. Cutoffs: ≥17 units for high binding and ≥12 units for intermediate binding.	Pretransplant and posttransplant sera. Posttransplant samples were obtained when available at five routine first-year clinic visits or during suspected rejection; posttransplant sera were available for 189 recipients.	NR.	DSA assessed by Luminex single-antigen bead assay. DSA groups: no DSA, 175; dnDSA, 19; DSA at transplant, 6.	Maintenance immunosuppression NR in the main methods. Rejection therapies included high-dose methylprednisolone, ATG for graft dysfunction, and possible plasmapheresis/IVIg for DSA.	AMR or CMR assessed by ISHLT biopsy grading; grades ≥2 included in analysis. Biopsy surveillance was weekly in month 1, every other week for the next 2 months, monthly for 3 months, every 2 months for 6 months, and when rejection was suspected; no routine biopsies after year 1. CAV determined by yearly angiography; detailed angiographic grading criteria NR.
Urban et al., 2016 [32]	Sandwich ELISA, One Lambda/Thermo Fisher; AT1R-precoated plates; 1:100 serum dilution. Cutoff 17 U/mL; positive >17 U/mL and negative ≤17 U/mL.	Two pretransplant samples: before HeartMate II implantation and at the time of HT.	100% HeartMate II LVAD; mean support 11 months, range 1–53 months.	Anti-HLA antibodies before HeartMate II implantation in 3/69 (4.3%). During support, 6/67 (9%) initially non-HLA-sensitized patients developed anti-HLA antibodies. At transplant: AT1R−/HLA−, 13; AT1R−/HLA+, 3; AT1R+/HLA−, 47; AT1R+/HLA+, 4.	ATG induction 1.5 mg/kg; maintenance cyclosporine or tacrolimus plus MMF plus steroids.	ACR ≥2R and pAMR of any grade were included in time-to-event analyses. Rejection identification/classification was based on EMB histopathology and immunohistochemistry according to ISHLT guidelines. CAV was not included as an outcome because only 41 coronary angiograms were available.
Chau et al., 2020 [37]	Sandwich ELISA, One Lambda. Main cutoff: >10 U/mL. Additional event analyses used >17 U/mL and >40 U/mL. Per manufacturer instructions, concentrations >40 U/mL were assigned 40 U/mL, with dilution used to obtain absolute values for correlation analyses.	Before LVAD, after LVAD, at HT, and after HT; 280 sera tested. Pre-LVAD sera were available in 65/77; repeated-measures four-timepoint subset included 52 patients.	100% continuous-flow LVAD bridge: axial 54/77 (70%) and centrifugal 23/77 (30%).	HLA typed at A, B, C, DR, and DQ by qPCR. Anti-HLA antibodies assessed by Luminex. Sensitization defined as cPRA >10%. HLA-DSA at transplant defined as MFI >1000. Positive DSA at transplant occurred in 6/77 (8%), all in the elevated AT1R-Ab group. De novo DSA after HT occurred in 5 patients.	Tacrolimus plus MMF plus steroid taper in all patients; induction in 36/77 (47%). No antibody desensitization before or after HT.	Primary endpoint: death, treated rejection, or CAV grade ≥2 by ISHLT. Treated rejection defined as ACR ≥2R, pAMR ≥1+, or biopsy-negative rejection.
Thohan et al., 2020 [31]	Commercial One Lambda/Thermo Fisher enzyme immunoassay; samples run in duplicate. Positive >17 U/mL; at-risk 10–17 U/mL; negative <10 U/mL.	Final crossmatch serum and archived posttransplant serum from routine clinical practice; both pre- and posttransplant status assessed. Pretransplant categories: negative 126, at-risk 58, positive 107. Posttransplant categories: negative 90, at-risk 40, positive 46.	LVAD before HT in 185/291 (64%). Pretransplant LVAD exposure by AT1R-Ab group: negative 69 (49%), at-risk 36 (62%), positive 92 (86%).	Baseline HLA sensitization in 63/291 (22%); cPRA >40% in 25 patients. DSA-specific details NR.	Tacrolimus plus MMF plus prednisone taper; induction rare, with <2% receiving thymoglobulin or basiliximab. Most patients were weaned from steroids by 6–12 months.	ACR graded using the ISHLT biopsy scoring system. AMR assessed by H&E only until 2010 and thereafter by H&E plus immunohistochemical staining for complement deposition. CAV presence/absence abstracted from the medical record using standard ISHLT definitions.
Moreno et al., 2022 [30]	AT1R-Ab testing performed at the UCLA HLA Laboratory; values reported at 1:100 dilution; commercial platform NR in the main text. Low 1–10 U/mL, intermediate 11–20 U/mL, and high >21 U/mL. Intermediate/high groups constituted the AT1R-Ab cohort (>10 U/mL), and the low-titer group served as the comparator (≤10 U/mL).	Clinically indicated post-HT testing for graft dysfunction; testing occurred at presentation of graft dysfunction or immediately before. Routine AT1R-Ab testing at the time of transplant was not standard practice.	8/11 (73%) in the AT1R-Ab cohort were bridged with LVAD.	Most patients had class I and II DSA MFI <500 immediately proximal to AT1R-Ab testing; no appreciable DSA. No canonical AMR, including negative C4d and CD68.	Tacrolimus, MMF, prednisone, and induction therapy were reported. Treatments for suspected AT1R-Ab-mediated rejection included ARB therapy, PLEX, IVIg, rituximab, and tocilizumab.	Inclusion required graft dysfunction without moderate ACR (>1R/1A) and without canonical AMR. Clinical graft dysfunction included heart failure symptoms or imaging evidence, including LVEF drop >10%, LVEF ≤45%, or new diastolic dysfunction. Pathologic HLA-AMR required >50% strong C4d staining and >10% intravascular CD68+ cells. Formal study-level CAV outcome definition NR; progressive CAV was considered clinically as an alternative explanation.

Abbreviations: ACR, acute cellular rejection; AMR, antibody-mediated rejection; ARB, angiotensin receptor blocker; AT1R, angiotensin II type 1 receptor; AT1R-Ab, angiotensin II type 1 receptor antibody; ATG, antithymocyte globulin; BiVAD, biventricular assist device; BPAR, biopsy-proven acute rejection; CAV, cardiac allograft vasculopathy; CMR, cellular-mediated rejection; CNI, calcineurin inhibitor; cPRA, calculated panel-reactive antibody; dnDSA, de novo donor-specific antibody; DSA, donor-specific antibody; ELISA, enzyme-linked immunosorbent assay; EMB, endomyocardial biopsy; H&E, hematoxylin and eosin; HLA, human leukocyte antigen; HT, heart transplantation; ISHLT, International Society for Heart and Lung Transplantation; IVIg, intravenous immunoglobulin; LVAD, left ventricular assist device; MCS, mechanical circulatory support; MFI, mean fluorescence intensity; MMF, mycophenolate mofetil; NR, not reported; pAMR, pathologic antibody-mediated rejection; PLEX, plasmapheresis; ROC, receiver operating characteristic.

## Data Availability

The data supporting the findings of this systematic review are available within the article and its Supplementary Materials. Additional details may be made available from the corresponding author upon reasonable request.

## Supplementary Materials

The following supporting information can be downloaded at: https://www.mdpi.com/article/10.3390/jcm15145419/s1, Table S1: Full database-specific search strategies; Table S2: Methodological quality and risk-of-bias assessment of included studies using the NIH Quality Assessment Tool; Table S3: PRISMA 2020 Checklist; Table S4: Excluded full-text studies and reasons for exclusion.

## Abbreviations

The following abbreviations are used in this manuscript:

ACE	angiotensin-converting enzyme;
ACR	acute cellular rejection;
AMR	antibody-mediated rejection;
ARB	angiotensin receptor blocker;
AT1R	angiotensin II type 1 receptor;
AT1R-Ab	angiotensin II type 1 receptor antibody;
AT2R	angiotensin II type 2 receptor;
CAV	cardiac allograft vasculopathy;
CMIT	change in maximal intimal thickness;
CMR	cellular-mediated rejection;
DSA	donor-specific antibody;
dnDSA	de novo donor-specific antibody;
ETAR	endothelin type A receptor;
HF	heart failure;
HLA	human leukocyte antigen;
HT	heart transplantation;
ICM	ischemic cardiomyopathy;
IVIG	intravenous immunoglobulin;
LVAD	left ventricular assist device;
MCS	mechanical circulatory support;
MICA	major histocompatibility complex class I chain-related antigen A;
NA	not available;
NIH	National Institutes of Health;
NICM	nonischemic cardiomyopathy;
OHT	orthotopic heart transplantation;
PRISMA	Preferred Reporting Items for Systematic Reviews and Meta-Analyses;
RAAS	renin–angiotensin–aldosterone system;
ROB	risk of bias;
TCAD	transplant coronary artery disease;
TRAS	transplant renal artery stenosis
