# Supplementary material for: Angiotensin II Type 1 Receptor Expression and Anti-AT1R Antibodies in Heart Transplantation: A Systematic Review of Distinct but Related Non-HLA Immune Pathways
Source: J Clin Med. 2026 Jul 10;15(14):5419. doi: 10.3390/jcm15145419 (PMC13410379; doi:10.3390/jcm15145419)
Supplement: Supplementary file 1 [file jcm-15-05419-s001.zip › jcm-4379629-supplementary.pdf]

Supplementary Table S1. Full database-specific search strategies.

Search date: December 2025. **Search concept:** angiotensin II type 1 receptor/anti-AT1R antibody terminology combined with heart transplantation.

Database	Full search strategy	Date searched	Records identified, n
PubMed	("Angiotensin II Type 1 Receptor"[Title/Abstract] OR "Angiotensin AT1 Receptor"[Title/Abstract] OR "Angiotensin Type 1 Receptor"[Title/Abstract] OR "Angiotensin II Type 1b Receptor"[Title/Abstract] OR "Angiotensin Type 1b Receptor"[Title/Abstract] OR "Angiotensin AT1b Receptor"[Title/Abstract] OR "Angiotensin II Type 1a Receptor"[Title/Abstract] OR "Angiotensin AT1a Receptor"[Title/Abstract] OR "Angiotensin Type 1a Receptor"[Title/Abstract] OR AT1R[Title/Abstract] OR "Angiotensin II Receptor"[Title/Abstract] OR "anti-AT1R"[Title/Abstract] OR "AT1R-Ab"[Title/Abstract] OR "AT1R antibody"[Title/Abstract] OR "angiotensin II type 1 receptor antibody"[Title/Abstract]) AND ("Heart Grafting"[Title/Abstract] OR "Heart Transplantation"[MeSH Terms] OR "Heart Transplantation"[Title/Abstract] OR "Cardiac Transplantation"[Title/Abstract] OR "heart transplant"[Title/Abstract] OR "orthotopic heart transplantation"[Title/Abstract])	December 2025	82
Scopus	TITLE-ABS-KEY(("Angiotensin II Type 1 Receptor" OR "Angiotensin AT1 Receptor" OR "Angiotensin Type 1 Receptor" OR "Angiotensin II Type 1b Receptor" OR "Angiotensin Type 1b Receptor" OR "Angiotensin AT1b Receptor" OR "Angiotensin II Type 1a Receptor" OR "Angiotensin AT1a Receptor" OR "Angiotensin Type 1a Receptor" OR AT1R OR "Angiotensin II Receptor" OR "anti-AT1R" OR "AT1R-Ab" OR "AT1R antibody" OR "angiotensin II type 1 receptor antibody") AND ("Heart Grafting" OR "Heart Transplantation" OR "Cardiac Transplantation" OR "heart transplant" OR "orthotopic heart transplantation"))	December 2025	107
Web of Science	TS=(("Angiotensin II Type 1 Receptor" OR "Angiotensin AT1 Receptor" OR "Angiotensin Type 1 Receptor" OR "Angiotensin II Type 1b Receptor" OR "Angiotensin Type 1b Receptor" OR "Angiotensin AT1b Receptor" OR "Angiotensin II Type 1a Receptor" OR "Angiotensin AT1a Receptor" OR "Angiotensin Type 1a Receptor" OR AT1R OR "Angiotensin II Receptor" OR "anti-AT1R" OR "AT1R-Ab" OR "AT1R antibody" OR "angiotensin II type 1 receptor antibody") AND ("Heart Grafting" OR "Heart Transplantation" OR "Cardiac Transplantation" OR "heart transplant" OR "orthotopic heart transplantation"))	December 2025	57
Cochrane Library	(("Angiotensin II Type 1 Receptor" OR "Angiotensin AT1 Receptor" OR "Angiotensin Type 1 Receptor" OR "Angiotensin II Type 1b Receptor" OR "Angiotensin Type 1b Receptor" OR "Angiotensin AT1b Receptor" OR "Angiotensin II Type 1a Receptor" OR "Angiotensin AT1a Receptor" OR "Angiotensin Type 1a Receptor" OR AT1R OR "Angiotensin II Receptor" OR "anti-AT1R" OR "AT1R-Ab" OR "AT1R antibody" OR "angiotensin II type 1 receptor antibody") AND ("Heart Grafting" OR "Heart Transplantation" OR "Cardiac Transplantation" OR "heart transplant" OR "orthotopic heart transplantation")) in title, abstract, or keyword	December 2025	48

Abbreviations: ACR, acute cellular rejection; AGTR1, angiotensin II receptor type 1 gene; AMR, antibody-mediated rejection; AT1R, angiotensin II type 1 receptor; AT1R-Ab, angiotensin II type 1 receptor antibody; HLA, human leukocyte antigen.

Note: A total of 294 records were identified across databases before duplicate removal. After removal of 138 duplicates, 156 unique records were screened. No additional eligible studies were identified after the expanded search terms were applied.

Supplementary Table S2. Methodological quality and risk-of-bias assessment of included studies using the NIH Quality Assessment Tool

This table summarizes the item-level methodological quality and risk-of-bias assessment of the included observational studies using the National Institutes of Health (NIH) Quality Assessment Tool for observational cohort and cross-sectional studies. Because the NIH tool is intended primarily for qualitative domain-based appraisal and does not provide a formal numerical scoring system, an author-defined, prespecified modified quantitative summary was applied to support transparent and reproducible reporting of item-level responses. Responses were summarized as follows: Yes = 1 point, No = 0.5 points, and NR/NA/CD = 0 points. Studies were categorized as good (11–14 points), fair (7.5–10.5 points), or poor (0–7 points). This score was used only as an author-defined summary aid and should not be interpreted as a validated NIH scoring system. Final ratings were interpreted qualitatively in the context of the NIH domains, including selection bias, exposure and outcome measurement, adequacy of follow-up, and adjustment for potential confounding.

Study ID	Q1	Q2	Q3	Q4	Q5	Q6	Q7	Q8	Q9	Q10	Q11	Q12	Q13	Q14	Total	Rating
Zagrosek	Yes	Yes	Yes	Yes	No	Yes	Yes	NA	Yes	Yes	Yes	NA	NR	NR	9.5	Fair
Gullestad 1998	Yes	Yes	Yes	Yes	No	NR	Yes	NA	Yes	Yes	Yes	NA	NR	NR	8.5	Fair
Yousufuddin 2004	Yes	Yes	Yes	Yes	Yes	No	Yes	No	Yes	Yes	Yes	NA	Yes	NR	11	Good
Moreno 2022	Yes	Yes	Yes	Yes	No	Yes	Yes	NA	Yes	No	Yes	NA	Yes	NR	9.5	Fair
Yousufuddin 2004 (1)	Yes	No	Yes	Yes	Yes	Yes	Yes	NA	Yes	Yes	Yes	NA	Yes	NR	10.5	Fair
Chau 2020	Yes	Yes	Yes	Yes	Yes	Yes	Yes	NA	Yes	Yes	Yes	NA	Yes	Yes	12	Good
Yamani 2006	Yes	Yes	Yes	Yes	Yes	Yes	Yes	NA	Yes	Yes	Yes	NA	Yes	Yes	12	Good
Reinsmoen 2014	Yes	Yes	Yes	Yes	Yes	Yes	Yes	NA	Yes	Yes	Yes	NA	Yes	Yes	12	Good
Hiemann 2012	Yes	Yes	Yes	Yes	Yes	Yes	Yes	NA	Yes	Yes	Yes	Yes	Yes	No	12.5	Good
Dieterlen 2014	Yes	Yes	Yes	Yes	Yes	No	Yes	NA	Yes	No	Yes	NA	Yes	Yes	11	Good
Thohan 2020	Yes	Yes	Yes	Yes	Yes	Yes	Yes	NA	Yes	Yes	Yes	Yes	Yes	Yes	13	Good
Urban 2016	Yes	Yes	Yes	Yes	Yes	Yes	Yes	NA	Yes	Yes	Yes	Yes	Yes	Yes	13	Good

NIH Quality Assessment Tool item key

Item	Assessment domain/question
Q1	Was the research question or objective in this paper clearly stated?
Q2	Were eligibility/selection criteria for the study population prespecified and clearly described?
Q3	Were the participants in the study representative of those who would be eligible for the test/service/intervention in the general or clinical population of interest?
Q4	Were all eligible participants that met the prespecified entry criteria enrolled?
Q5	Was the sample size sufficiently large to provide confidence in the findings?
Q6	For the analyses in this paper, were the exposure(s) of interest measured prior to the outcome(s) being measured?
Q7	Was the time frame sufficient so that one could reasonably expect to see an association between exposure and outcome if it existed?
Q8	For exposures that can vary in amount or level, did the study examine different levels of the exposure as related to the outcome (eg, categories of exposure, or exposure measured as continuous variable)?
Q9	Were the exposure measures (independent variables) clearly defined, valid, reliable, and implemented consistently across all study participants?
Q10	Was the exposure(s) assessed more than once over time?
Q11	Were the outcome measures prespecified, clearly defined, valid, reliable, and assessed consistently across all study participants?
Q12	Were the people assessing the outcomes blinded to the participants' exposures/interventions?
Q13	Was the loss to follow-up after baseline 20% or less? Were those lost to follow-up accounted for in the analysis?
Q14	Were key potential confounding variables measured and adjusted statistically for their impact on the relationship between exposure(s) and outcome(s)?

Abbreviations: CD, cannot determine; NA, not applicable; NIH, National Institutes of Health; NR, not reported; Q, question/item.

Supplementary Table S3. PRISMA 2020 Checklist

Section	Item	PRISMA 2020 checklist item	Reported location in manuscript	Comments / completion notes
TITLE	1	Identify the report as a systematic review.	Title page	The manuscript title identifies the article as a systematic review.
ABSTRACT	2	See the PRISMA 2020 for Abstracts checklist.	Abstract	Structured abstract includes background, aim, methods, results, and conclusions.
INTRODUCTION	3	Describe the rationale for the review in the context of existing knowledge.	Introduction	Rationale provided for reviewing A.T.1R expression and A.T.1R-Ab in HTx due to heterogeneous prior evidence.
INTRODUCTION	4	Provide an explicit statement of the objective(s) or question(s) the review addresses.	Abstract; Introduction	Objective stated as systematically evaluating evidence linking A.T.1R gene expression and A.T.1R-Ab status with post-HTx outcomes.
METHODS	5	Specify the inclusion and exclusion criteria for the review and how studies were grouped for the syntheses.	Methods 2.2	Eligibility criteria specify study designs, population, exposure/concept, outcomes, language, full-text status, and excluded publication types.
METHODS	6	Specify all databases, registers, websites, organisations, reference lists and other sources searched or consulted to identify studies. Specify the date when each source was last searched or consulted.	Methods 2.1	Scopus, PubMed, Cochrane Library, and Web of Science searched in December 2025; references of included studies checked.
METHODS	7	Present the full search strategies for all databases, registers and websites, including any filters and limits used.	Methods 2.1; Supplementary Table S1	Core Boolean strategy reported in the manuscript; database-specific strategies reported in Supplementary Table S1.
METHODS	8	Specify the methods used to decide whether a study met inclusion criteria, including how many reviewers screened each record and each report, whether they worked independently, and how disagreements were resolved.	Methods 2.3	Two authors independently screened titles/abstracts and full texts; disagreements resolved by discussion/consensus.
METHODS	9	Specify the methods used to collect data from reports, including how many reviewers collected data, whether they worked independently, processes for obtaining/confirming data, and any automation tools used.	Methods 2.3	Data extraction by MM and AH using standardized criteria. No automation tools or author-contact procedures reported.
METHODS	10a	List and define all outcomes for which data were sought. Specify whether all results compatible with each outcome domain were sought.	Methods 2.2; Methods 2.3	Outcomes included ACR, AMR, CAV, graft dysfunction, survival, freedom from adverse events, and TCAD progression.
METHODS	10b	List and define all other variables for which data were sought. Describe any assumptions made about missing or unclear information.	Methods 2.3; Tables 1-3	Variables included author, year, country, study design, participant demographics, sample size, assay characteristics, timing, LVAD/MCS exposure, HLA-DSA/HLA context, immunosuppression, rejection definitions, and CAV definitions.
METHODS	11	Specify the methods used to assess risk of bias in the included studies, including details of tools used, number of reviewers, independent assessment, and disagreement resolution.	Methods 2.4; Supplementary Table S2	NIH Quality Assessment Tool used by two independent reviewers; modified scoring approach described; disagreements resolved by discussion/consensus.
METHODS	12	Specify for each outcome the effect measure(s) used in the synthesis or presentation of results.	Results 3.5-3.9; Tables 1-3	Narrative synthesis. Study-specific measures were reported when available, including prevalence, percentages, HRs, confidence intervals, P values, correlations, and event-free survival.
METHODS	13a	Describe the processes used to decide which studies were eligible for each synthesis.	Methods 2.2; Results 3.4-3.9	Studies grouped narratively by exposure type and outcome: A.T.1R mRNA expression, A.T.1R-Ab prevalence, ACR, AMR, survival, and CAV.
METHODS	13b	Describe any methods required to prepare the data for presentation or synthesis.	Methods 2.3; Tables 1-3	Extracted variables were standardized across evidence tables; NR/NA used where variables were not reported or not applicable.
METHODS	13c	Describe any methods used to tabulate or visually display results of individual studies and syntheses.	Tables 1-3; Figure 1	Study characteristics, baseline variables, and antibody-methodology heterogeneity presented in tables; study selection shown with PRISMA flow diagram.

Section	Item	PRISMA 2020 checklist item	Reported location in manuscript	Comments / completion notes
METHODS	13d	Describe any methods used to synthesize results and provide rationale for the choice(s).	Results 3.4-3.9; Discussion	Narrative synthesis used because of heterogeneity in assay platforms, cutoffs, timing, outcomes, and study designs. No meta-analysis performed.
METHODS	13e	Describe methods used to explore possible causes of heterogeneity among study results.	Table 3; Results 3.5; Discussion	Methodological heterogeneity summarized by assay type, cutoff, sampling timing, LVAD/MCS exposure, HLA context, immunosuppression, and endpoint definitions.
METHODS	13f	Describe any sensitivity analyses conducted to assess robustness of synthesized results.	Not applicable / not performed	No quantitative meta-analysis or formal sensitivity analysis was performed.
METHODS	14	Describe any methods used to assess risk of bias due to missing results in a synthesis.	Not reported / not performed	No formal reporting-bias or small-study-effect assessment was performed.
METHODS	15	Describe any methods used to assess certainty or confidence in the body of evidence for an outcome.	Methods 2.4; Results 3.3; Discussion	Certainty was not assessed with GRADE. Methodological quality/risk of bias was assessed with NIH tool and considered in the interpretation.
RESULTS	16a	Describe the results of the search and selection process, from records identified to studies included, ideally using a flow diagram.	Results 3.1; Figure 1	294 records identified; 138 duplicates removed; 156 screened; 17 full-text reports assessed; 6 excluded; 12 studies included.
RESULTS	16b	Cite studies that might appear to meet inclusion criteria but were excluded, and explain why they were excluded.	Results 3.1; Supplementary Table S4	Excluded full-text articles and reasons are reported in Supplementary Table S4.
RESULTS	17	Cite each included study and present its characteristics.	Results 3.2; Table 1; Table 2	Characteristics of the 12 included studies are summarized in Tables 1 and 2.
RESULTS	18	Present assessments of risk of bias for each included study.	Results 3.3; Supplementary Table S2	Overall quality summarized as 8/12 good and 4/12 fair; complete study-level assessment in Supplementary Table S2.
RESULTS	19	For all outcomes, present for each study summary statistics and effect estimates, with confidence/credible intervals where applicable.	Results 3.4-3.9; Tables 1-3	Narrative results include study-specific prevalence, event rates, HRs, P values, correlations, survival/adverse-event estimates, and qualitative conclusions where available.
RESULTS	20a	For each synthesis, briefly summarize the characteristics and risk of bias among contributing studies.	Results 3.2-3.3; Tables 1-3	Study designs and methodological quality summarized before outcome-specific synthesis; heterogeneity summarized in Table 3.
RESULTS	20b	Present results of all statistical syntheses conducted.	Not applicable	No statistical meta-analysis was conducted.
RESULTS	20c	Present results of investigations of possible causes of heterogeneity among study results.	Results 3.5; Table 3; Discussion	Heterogeneity reported descriptively across antibody assays, thresholds, timing, LVAD/MCS exposure, HLA/DSA context, and endpoint definitions.
RESULTS	20d	Present results of all sensitivity analyses conducted to assess robustness of synthesized results.	Not applicable	No sensitivity analyses were performed.
RESULTS	21	Present assessments of risk of bias due to missing results for each synthesis assessed.	Not reported / not performed	No formal assessment of bias due to missing results was conducted.
RESULTS	22	Present assessments of certainty or confidence in the body of evidence for each outcome assessed.	Results 3.3; Discussion	Formal GRADE certainty assessment not performed; risk-of-bias findings and evidence limitations discussed.
DISCUSSION	23a	Provide a general interpretation of the results in the context of other evidence.	Discussion; Conclusion	Discussion interprets A.T.1R-Ab and A.T.1R expression findings in context of non-HLA antibody literature and transplant injury mechanisms.
DISCUSSION	23b	Discuss any limitations of the evidence included in the review.	Discussion	Limitations include small sample sizes, heterogeneity, limited confounder adjustment, variable assay thresholds, and lack of standardized methods.
DISCUSSION	23c	Discuss any limitations of the review processes used.	Discussion; Methods 2.4	Review limitations include narrative synthesis, protocol not registered, and lack of quantitative pooling or formal certainty assessment.
DISCUSSION	23d	Discuss implications of the results for practice, policy, and future research.	Discussion; Future Research and Implications; Conclusion	Future studies, standardized A.T.1R-Ab testing, clinically meaningful cutoffs, risk stratification, and targeted therapeutic trials are discussed.
OTHER INFORMATION	24a	Provide registration information for the review, including register name and registration number, or state that the review was not registered.	Methods 2; Protocol statement	The manuscript states that the protocol was not registered.

Section	Item	PRISMA 2020 checklist item	Reported location in manuscript	Comments / completion notes
OTHER INFORMATION	24b	Indicate where the review protocol can be accessed or state that a protocol was not prepared.	Methods 2	No registered protocol was prepared/provided; rationale for not registering retrospectively is stated.
OTHER INFORMATION	24c	Describe and explain any amendments to information provided at registration or in the protocol.	Not applicable	No protocol registration; therefore, no protocol amendments reported.
OTHER INFORMATION	25	Describe sources of financial or non-financial support for the review and the role of funders/sponsors.	Funding statement	The manuscript states that the research received no external funding.
OTHER INFORMATION	26	Declare any competing interests of review authors.	Conflicts of Interest statement	The manuscript states that the authors declare no conflicts of interest.
OTHER INFORMATION	27	Report which data, analytic code, and other materials are publicly available and where they can be found.	Data Availability Statement; Supplementary materials	The manuscript states that data supporting the review are available within the article and supplementary materials, with additional details available from the corresponding author upon reasonable request.

Abbreviations: ACR, acute cellular rejection; AMR, antibody-mediated rejection; A.T.1R-Ab, angiotensin II type 1 receptor antibody; CAV, cardiac allograft vasculopathy; CMR, cellular-mediated rejection; DSA, donor-specific antibody; HTx, heart transplantation; LVAD, left ventricular assist device; PRISMA, Preferred Reporting Items for Systematic Reviews and Meta-Analyses; TCAD, transplant coronary artery disease.

Supplementary Table S4. Full-text articles were excluded after eligibility assessment

Study	Year	Reason for Full-Text Review	Reason for Exclusion
Opelz et al.	2006	Evaluated ACE inhibitor/ARB therapy in kidney and heart transplant recipients	Did not assess AT1R antibodies; focused on pharmacologic ACEI/ARB treatment and transplant outcomes.
Richter et al.	2003	Experimental study of losartan/enalapril and transplant vasculopathy	Animal model study; no human heart transplant recipients and no assessment of AT1R antibodies.
Urban et al.	2016	AT1R antibodies in HeartMate II LVAD recipients	LVAD/mechanical circulatory support cohort; not a heart-transplant recipient cohort evaluating post-transplant outcomes.
Zhang et al.	2018	AT1R antibody development after mechanical circulatory support implantation	Primarily MCS/LVAD/TAH population; not designed to evaluate AT1R antibodies in heart-transplant recipients.
Oaks et al.	2018	Analytical study of anti-AT1R assay specificity	Laboratory assay-validation study; did not evaluate clinical heart-transplant outcomes or AT1R-associated rejection.
